# A novel synthesis method of cyclopentadecanone and cyclopentadecanolide from vegetable oil

**DOI:** 10.1186/s13065-022-00840-y

**Published:** 2022-06-22

**Authors:** Pin Liu, Weiguang Li, Xiongmin Liu

**Affiliations:** 1 College of Chemistry and Chemical Engineering, Guangxi University, Nanning, 530004 Guangxi China; 2 Department of Science and Technology, Guangxi University for Nationalities, Nanning, 530006 China

**Keywords:** *Malania oleifera Chum* oil, Cyclopentadecanone, Cyclopentadecanolide, Synthesis

## Abstract

*Malania oleifera Chum* is a unique renewable plant resource in China, its fruit oil has a high content of 15-tetracosenic acid, and it is a good raw material for the synthesis of cyclopentadecanone and cyclopentadecanolide. A Novel synthesis method of cyclopentadecanone and cyclopentadecanolide from vegetable oil was designed, and the synthesis yields of c cyclopentadecanone and cyclopentadecanolide were 38.5% and 63.0%, respectively. The effect of different ester groups on cyclization of α,ω-difatty acid alkyl ester in cyclopentadecanone synthesis, and effect of catalysts on cyclization ofω-hydroxycarboxylic acid triglyceride in cyclopentadecanolide synthesis were investigated. The designed synthesis strategy has the characteristics of less synthesis steps and high utilization efficiency of 15-tetracosenic acid, which has simple and economic.

**Supplementary Information:**

The online version contains supplementary material available at 10.1186/s13065-022-00840-y.

## Introduction

Macrocyclic musk is an expensive flavor, it widely used in perfumes, cosmetics, food, and medicine. Cyclopentadecanolide of macrocyclic lactones and cyclopentadecanone of macrocyclic ketoneare two important macrocyclic musk [1], they constitute an important class of natural products possessing diverse biological activities, like antibiotic, antitumour etc. [2]. Cyclopentadecanone was evaluated for genotoxicity, repeated dose toxicity, reproductive toxicity, local respiratory toxicity, photoallergenicit, skin sensitization, and environmental safety. Data show that cyclopentadecanone is not genotoxic [3]. Cyclopentadecanolide were tested for genotoxicity, the results show that it is not genotoxic [4], and it has been approved by Food and Drug Administration (FDA) of America for use in food [5]. Cyclopentadecanolide exists in many plants [6, 7], but its content is very low, this is difficult to extract and separate cyclopentadecanolide from natural plants. The commercialization cyclopentadecanolide mainly comes from chemical synthesis methods. Chemical synthesis of cyclopentadecanolactone is a common production method, several preparation methods have been developed [8–11]. Ookoshi [12] has been reported that the macrolactonization of x-hydroxyalkanoic acid in a highly concentrated solution is catalyzed by dealuminated HY zeolite. The HZSM-5 zeolite is one of the solid acids which have been widely used as viable alternatives to conventional acids in esterification reactions [13–15]. Lai [16] has been reported that macrolactonization of methyl 15-hydroxypentadecanoate to cyclopentadecanolide over Mo-Fe/HZSM-5 catalyst. The important intermediate of synthetic cyclopentadecanolide is 15-hydroxyalkanoic acid. However, the manufacture of 15-hydroxyalkanoic acid is complex and difficult when synthesized by chemical methods. Cyclopentanone is also a widely used macrocyclic musk compounds, cyclopentadecanone occurs, along with cyclopentadecanol in the secretion of the North American musk rat, as a natural product, its produces very little. There are many synthetic methods of cyclopentanone, McGinty reported the fragrance material review on cyclopentadecanone [17]. Many synthetic routes of cyclopentadecanone have been reported [18–23]. The common feature of cyclopentadecanolide and cyclopentadecanone is that they are a macrocyclic compound with 15 carbon atoms. The synthesis methods of 15-hydroxypentadecarboxylic acid and1, 15-pentadecanedioate is complex, if chemical synthesis is used from petroleum products. The synthesis of cyclopentadecanolide and cyclopentadecanone from biological resources is worthy of attention. Rana [24] and Sytniczuk et al. [25] reported on study synthesis of macrocyclic lactones and ketones using biomass. Yao et al. [26] synthesized macrocyclic musk compounds from rapeseed oil. Erucic acid is the main component in rapeseed oil. In the synthesis of cyclopentadecanolide and cyclopentadecanone, the carbon chain needs to be increased, and the synthetic route is long.

*Malania oleifera Chun *and* S. K. Lee* (simple name: *Malania oleifera Chum*) is a wild woody plant, mainly distributed in Guangxi and Yunnan Province of China [27]. Its fruits contains oils and fats [28] (50–60% by weight) in which the main component is 15-tetracosenoic acid (nervonic acid) (45–55%), erucic acid (15–25%) and oleic acid (20–30%). The aforementioned compound is a good candidate for synthesizing macrocyclic musks, such as Guo et al. [29] synthesized cyclopentadecanolide using 15-tetracosenoic acid. The key step of this synthesis method is to separate and purify compound 15-tetracosenoic from mixed fatty acids of *Malania oleifera Chum*. However, it is difficult to separate and purify 15-tetracosenoic acid from the fatty acids mixture [30], and the yield of 15-tetracosenoic acid is only 15%, the utilization rate of 15-tetracosenoic acid is very low. Therefore, the efficient utilization of 15-tetracosenoic acid has theoretical significance and practical application value.

In the present work, our purpose is to make effective use of natural plant resources and improve the utilization rate of 15 tetraenoic acid in *Malania oleifera Chum* oil, to find a simple and novel method to synthesize cyclopentadecanolide and cyclopentadecanone. The synthesis of macrocyclic compounds Cyclopentanol and cyclopentanone directly from maleic acid model oil is a new exploration. The unique feature of the synthetic method is that it does not need to convert oil into fatty acids and separate 15-tetracosenoic acid from mixed fatty acids, and its synthesis steps are few, the process route is short and the yield is high. This is very meaningful for the efficient utilization of renewable resources.

## Materials and methods

### Materials and apparatus

*Malania oleifera Chun *and* S. K. Lee* (simple name: *Malania oleifera Chum*) has been collected by the herbarium of Guangxi Institute of Botany, China, No.: IBK00373464. *Malania oleifera Chum* oil was extracted from fruit of *Malania oleifera Chun* harvested in Guangxi province of China. Plant species identified by Professor Lai Jiaye, botany expert (College of Forestry, Guangxi University). The consists of oils and fats 53.2%(w/w), in which the fats mainly contain 15-tetracosenic acid 46.7%, 9-octadecenoic acid 27.9%, and erucic acid 12.5%, other fatty acids 12.9%.

Standard cyclopentadecanone was purchased from Aldrich Co. (USA), purity: 98.0%. Standard cyclopentadecanolide was purchased from Askrich Chemical Company Inc. (Japan), purity: 98%. In order to In order to further verify the correctness of the synthetic product, the spectra (IR, GC-MS and NMR) of the synthetic product and the standard were compared. Hexane, Shanghai Adamas-β Co. (China), purity: 97.0%. Acetic acid, Shanghai Adamas-β Co. (China), purity: 99.5%.

IR spectra were recorded by a SHIMAZHU FT-IR8400S spectrometer (Japan). Mass spectra were determined by SHIMAZHU GC-MS/QP5050A (Japan). NMR spectra were recorded by AVANCE III HD 600 NMR (Bruker, Switizerland).

### **Extraction of*Malania oleifera Chum*** oil and preparation of fatty acid methyl esters

The extraction followed Soxhlet method using 50 g sample of *Malania oleifera Chum*. It included the crushed *Malania oleifera Chum* and 8 h Soxhlet reflux in petroleum ether of boiling range 60–80 ℃. Preparation of fatty acid methyl esters used a slightly modified method based on Simoneau and Vicario et al. [31, 32].

### Analysis of reaction products

SHIMAZHU GC-MS/QP5050A (Japan) equipped with USA J&W Co. DB-1 column (30.0 m × 0.25 mm × 0.25 m) was used for compound identification. Helium was employed as carrier gas at a constant flow rate of 1.5 mL min^− 1^. Initial oven temperature was set at 150 ℃, held for 1 min, ramped at 4 ℃ min^− 1^ to 270 ℃ and held for further 5 min with heated capillary transfer line maintained at 270 ℃. Splitless injection was carried out at 270 ℃ and 0.2 µL of sample was injected. In the GC/MS full scan mode, *m*/*z* 40 to *m*/*z* 450 was recorded. Chromatographic peaks were identified with NIST mass spectral data library and the retention times were compared with standard compounds listed in NIST 2008 Mass Spectral Libraries V2.2 (Additional file 1).

### Preparation of cyclopentadecanone

A solution of *Malania olceifera* Chum oil (40.0 g) in hexane (300 mL) and acetic acid (90 mL) was ozonized at 0 ℃ for 4 h, and then H_2_O_2_ (30%, 40 mL) was added dropwisely for another 3 h period at room temperature. The reaction mixture was added to ice water, filtered and washed with water to obtain solid. The solid was dried to give 30 g mixed products P1.

P1 30 g, Methanol (270 g), and sulfuric acid (6 g) was mixed and refluxed for 4 h. The mixture was cooled, extracted with diethyl ether(100 mL 2 times), distilled to provide 30 g mixture of products P2.

P2 30 g in xylene (40 mL) was added to pulverized sodium (10 g) in refluxing xylene (500 mL) under nitrogen during 1 h. The mixture was refluxed for 1 h. Then, 150 mL ethanol was added slowly to the reactor at 80 ℃, after cooling to room temperature. Acetic acid (100 mL) was added to reactor, followed by 150 mL water. The xylene solution was separated from reaction mixture, and then treated with water. Xylene was evaporated under reduced pressure. The residue was distilled to give 16.0 g acyloin.

Hydrochloric acid (16 mL) was added to the mixture of crude acyloin (16.0 g) and Zn powder (4 g) during 1 h at 110 ℃. After the reaction maintained for another 30 min, the mixture was cooled to room temperature, extracted with benzene (200 mL), and washed with water.

The mixture was distilled, separated by vacuum distillation (or vacuum distillation of glycerin as entrainer) and recrystalled with ethanol to obtain cyclopentadecanone 16.0 g with purity of 97.4%.

### Preparation of cyclopentadecanolide

A solution of Malania olceifera Chum oil (200.0 g) in hexane (200 mL) and ethanol (200 mL) was ozonized at 0 ℃ for 4 h, and then reactant was added dropwisely 50 g potassium borohydride was dissolved in 500 mL aqueous solution at 10 ℃ for 3 h. Then neutralize to neutrality with hydrochloric acid for 2 h, filtration, washing with water for 3 times, and drying to obtain ω- hydroxycarboxylic acid triglyceride 135 g.

Put ω- hydroxycarboxylic acid triglyceride (5) (100 g) and catalyst into 1000 mL three bottles, then slowly add glycerol and heating up to make glycerol distillate at vacuum degree is < 755 mmHg, and mixture products 75 g was obtained when reaction is 40 h. The mixture was fractionating by distillation and recrystallized by ethanol, and cyclopentadecanolide 47 g were obtained.

### Structural characterization of cyclopentadecanone and cyclopentadecanolide

The structures of cyclopentadecanone and cyclopentadecanolide were determined by IR (KBr), GC-MS, ^1^H NMR (600 MHz, CDCl_3_) and ^13^C NMR (600 MHz, CDCl_3_), and compared with their standards.

## Results and discussion

### Synthetic strategy of cyclopentadecanone and cyclopentadecanolide

**Fig. 1:**
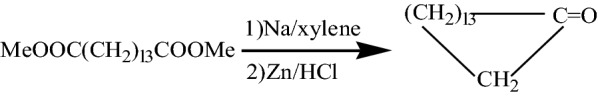
typical preparation method of cyclopentadecanone

Although there are many preparation methods for cyclopentadecanone [18, 19], the key reaction step is shown in Fig. 1.

**Fig. 2:**
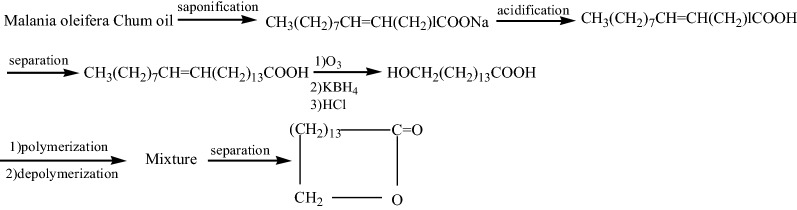
preparation method of cyclopentadecanolide from *Malania oleifera Chum* oil. l = 9, 11, 13 et al.

Therefore, the most important difficulty in the synthesis of cyclopentanone is how to obtain raw material MeOOC(CH_2_)_13_COOMe or HOOC(CH_2_)_13_COOH. Cyclopentadecanolide and cyclopentadecanone have similar carbon structure. Its commercial synthesis method is based on 15-hydroxypentadecarboxylic acid [8, 9]. Guo et al. [29] prepared 15-pentahydroxypentadecanoic acid from *Malania oleifera Chum* oil (1), and synthesized cyclopentadecanolide. The synthetic route is shown in Fig. 2.

Although on chemical synthesis, the preparation of mixed fats of is simple from *Malania oleifera Chum* oil, but separation of 15-tetracosenic acid is difficult to use crystallization method, because15-tetracosenic acid, 9-octadecenoic acid and erucic acid properties are similar in mixed fats. The yield of 15-tetracosenic acid (purity 95%) is only about 10% from *Malania oleifera Chum* oil.

**Fig. 3a:**
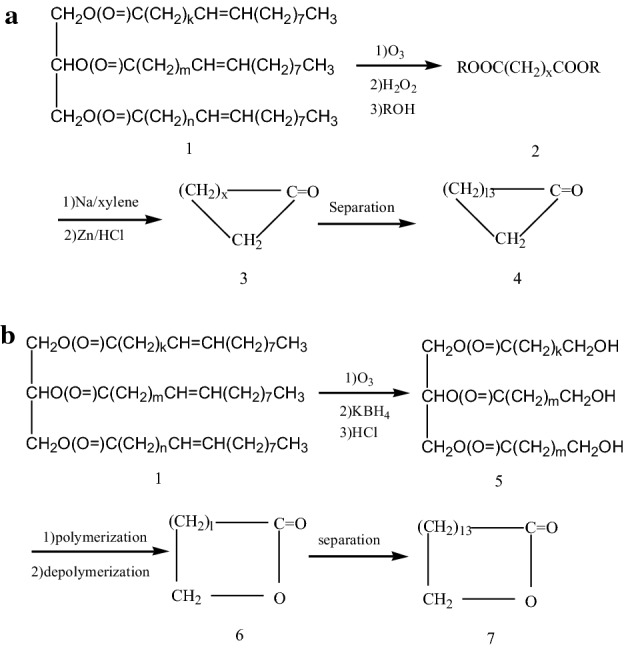
Synthesis strategy of cyclopentadecanone from Malania oleifera Chum oil. x = k, m, n; x = 9, 11, 13 et al. **b** Synthesis strategy of cyclopentadecanolide from Malania oleifera Chum oil. x = k, m, n; x = 9, 11, 13 et al.

In order to make efficient use of *Malania oleifera Chum* oil (1) resources and shorten the synthesis route of cyclopentadecanone (4) and cyclopentadecanolide (7), we proposed a synthesis strategy, as shown in Fig. 3a, b.

### Preparation of cyclopentadecanone

**Fig. 4:**
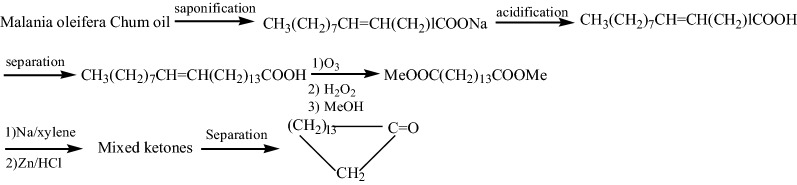
A typical synthesis route of cyclopentadecanolide from *Malania oleifera Chum* oil. l = 9, 11, 13 et al.

Firstly, we conducted a typical synthesis method experiment, and the synthesis route is shown in Fig. 4.

Since the yield of 15-tetracosenic acid with a purity of more than 90% obtained from *Malania oleifera Chum* oil is low, the yield of cyclopentadecanone is only 12.8%.

Then, we carried out the synthesis experiment according to the synthesis strategy (Fig. 3a). It is noteworthy that in Fig. 3a, when group Me is replaced by other groups, does it affect the yield? The effect of groups on the yield of cyclopentadecanone was investigated and the result was shown in Table 1.

**Table 1:** The effect of groups on the yield

Ester groups	Yields^a^ of cyclopentadecanone (%)
methyl	32.7
ethyl	38.5
propyl	31.1
butyl	21.0
typical synthetic route	12.8

^a^It is the yield calculated based on 15-tetracosenic acid as raw material

Table 1 show that the yield of cyclopentadecanone from ethyl ester was the highest (38.5%) among these ester groups. If it is compared with the typical synthetic route, the yield of the new synthetic strategy is about three times that of the typical synthetic route. Therefore, when cyclopentadecanone is synthesized according to the synthesis strategy, the utilization rate of nerve acid in *Malania oleifera Chum* oil is high.

Comparing the prepared cyclopentadecanone with the standard cyclopentadecanone, IR (KBr), GC-MS, ^1^H NMR (600 MHz, CDCl_3_) and ^13^C NMR (600 MHz, CDCl_3_) are identical.

Cyclopentadecanone is white solid, m.p. 60–61 ℃. IR (KBr): 3411, 2928, 2855, 1710, 1459, 1446, 1408, 1367, 1286, 1260, 1215, 1210, 1152, 1126, 1078, 729, 720, 570 cm^− 1^. ^1^H NMR (500 MHz, Chloroform-*d*) δ 2.43 (t, *J* = 6.7 Hz, 1 H), 1.65 (q, *J* = 6.7 Hz, 1 H), 1.43–1.32 (m, 2 H), 1.31 (dd, *J* = 6.4, 2.6 Hz, 3 H). ^13^ C NMR (126 MHz, Chloroform-*d*) δ 212.81, 212.81, 42.10, 27.58, 26.78, 26.73, 26.66, 26.44, 26.30, 23.46. GC-MS (EI): *m/z* = 224.4 (M^+^).

### Preparation of cyclopentadecanolide

*Malania oleifera Chum* is a renewable plant resource, and using its oil to synthesize cyclopentadecanolide has good application value.When cyclopentadecanolide was synthesized according to the preparation method of Fig. 2, its total yield was only 15.3%, and active ingredient utilization rate is low.

In order to make efficient use of resources, we designed a synthesis strategy, in the synthesis strategy (Fig.3b), alkali catalyst is very important, so the catalyst effect was investigated, and the result was shown in Table 2.

**Table 2:** Effect of catalysts on yields of cyclization

catalysts on	Yields of cyclopentadecanolide (%)^a^
NaOH	37.0
CH_3_ONa	52.0
CH_3_ONa/ NaOH	63.0
Preparation method of Fig. 2	15.3

^a^It is the yield calculated based on 15-tetracosenic acid as raw material

Table 2 shows that the yield of cyclopentadecanolide is 63% when using mixed catalyst CH_3_ONa/ NaOH, it is high compared with catalyst single NaOH or CH_3_ONa. Compared with the synthesis route in Fig. 2, the yield of the new synthesis strategy is 4.1 times that of the synthesis method in Fig. 2. Therefore this synthesis strategy is short and has the value of industrial application.

Comparing the prepared cyclopentadecanolide with the standard cyclopentadecanolide, IR (KBr), GC-MS, ^1^H NMR (500 MHz, CDCl_3_) and ^13^C NMR (126 MHz, CDCl_3_) are identical.

Cyclopentadecanolide is white solid, m.p. 36–37 ℃. IR (KBr): 3468, 2925, 2856, 2685, 1738, 1462, 1377, 1350, 1285, 1248, 1234, 1166, 1109, 1071, 1061, 1054, 1014, 963, 879, 723 cm^− 1^. ^1^H NMR (500 MHz, Chloroform-*d*) δ 4.18–4.11 (m, 1 H), 2.38–2.30 (m, 1 H), 1.65 (dddd, *J* = 19.1, 11.3, 7.8, 3.9 Hz, 2 H), 1.49–1.39 (m, 1 H), 1.35 (dd, *J* = 6.0, 2.7 Hz, 5 H). ^13^C NMR (126 MHz, Chloroform-*d*) δ 174.05, 63.98, 34.42, 28.38, 27.76, 27.13, 27.10, 26.90, 26.66, 26.34, 26.03, 25.92, 25.84, 25.11, 24.93. GC-MS (EI): *m/z* = 240.2 (M^+^).

## Conclusions

Musk compounds cyclopentadecanone and cyclopentadecanolide were synthesized from *Malania oleifera Chum* oil of a renewable plant resource. A novel synthesis method of cyclopentadecanone and cyclopentadecanolide were developed. Preparation method of cyclopentadecanone is three steps process which consists of ozonization, oxidation and esterification, and total yield is 38.5% when in terms of 15-tetracosenic acid. The effects of ester groups on cyclopentadecanone were explored. Preparation method of cyclopentadecanolide is three steps process which consists of ozonization and reduction reaction, cyclization, separation and 63% yield of cyclopentadecanolide was obtained. The effect of catalysts on cyclization of ω-hydroxycarboxylic acid triglyceride was investigated. The synthesis strategy is a very short technological route, and it is easy to industrialize.

## Supplementary information

**Additional file 1:** Appendix. Ms of cyclopentadecanolide and cyclopentadecanone.

## Data Availability

All data generated or analysed during this study are included in this published article.
