# Supplementary material for: A novel synthesis method of cyclopentadecanone and cyclopentadecanolide from vegetable oil
Source: BMC Chem. 2022 Jun 22;16(1):46. doi: 10.1186/s13065-022-00840-y (PMC9219129; doi:10.1186/s13065-022-00840-y)
Supplement: Supplementary file 1 — Additional file 1: Appendix. Ms of cyclopentadecanolide and cyclopentadecanone. [file 13065_2022_840_MOESM1_ESM.doc]

**Appendix**

**Cyclopentadecanolide**


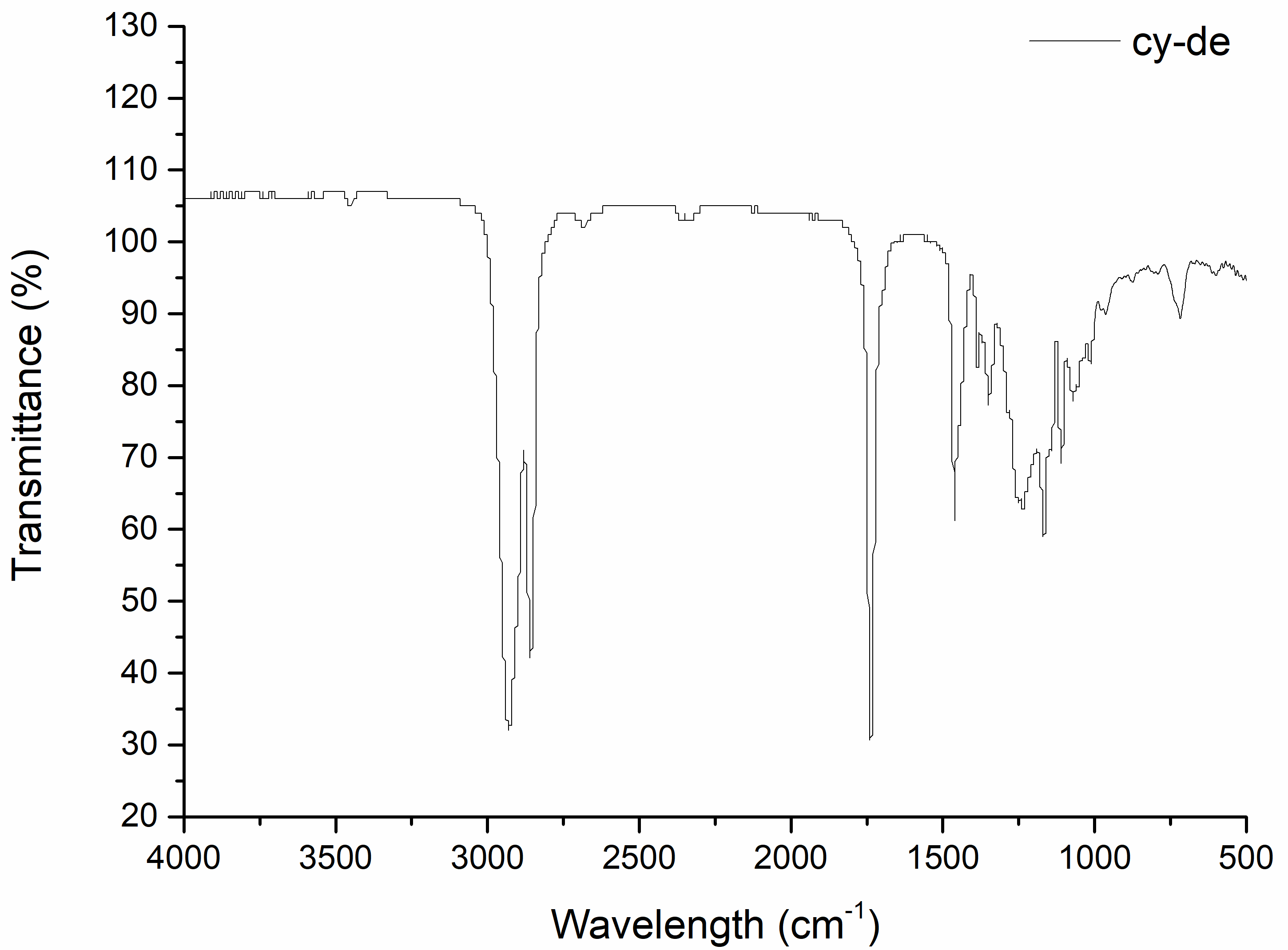


IR of cyclopentadecanolide

IR (KBr): 3468, 2925, 2856, 2685, 1738, 1462, 1377, 1350, 1285, 1248, 1234, 1166, 1109, 1071, 1061, 1054, 1014, 963, 879, 723 cm^-1^.


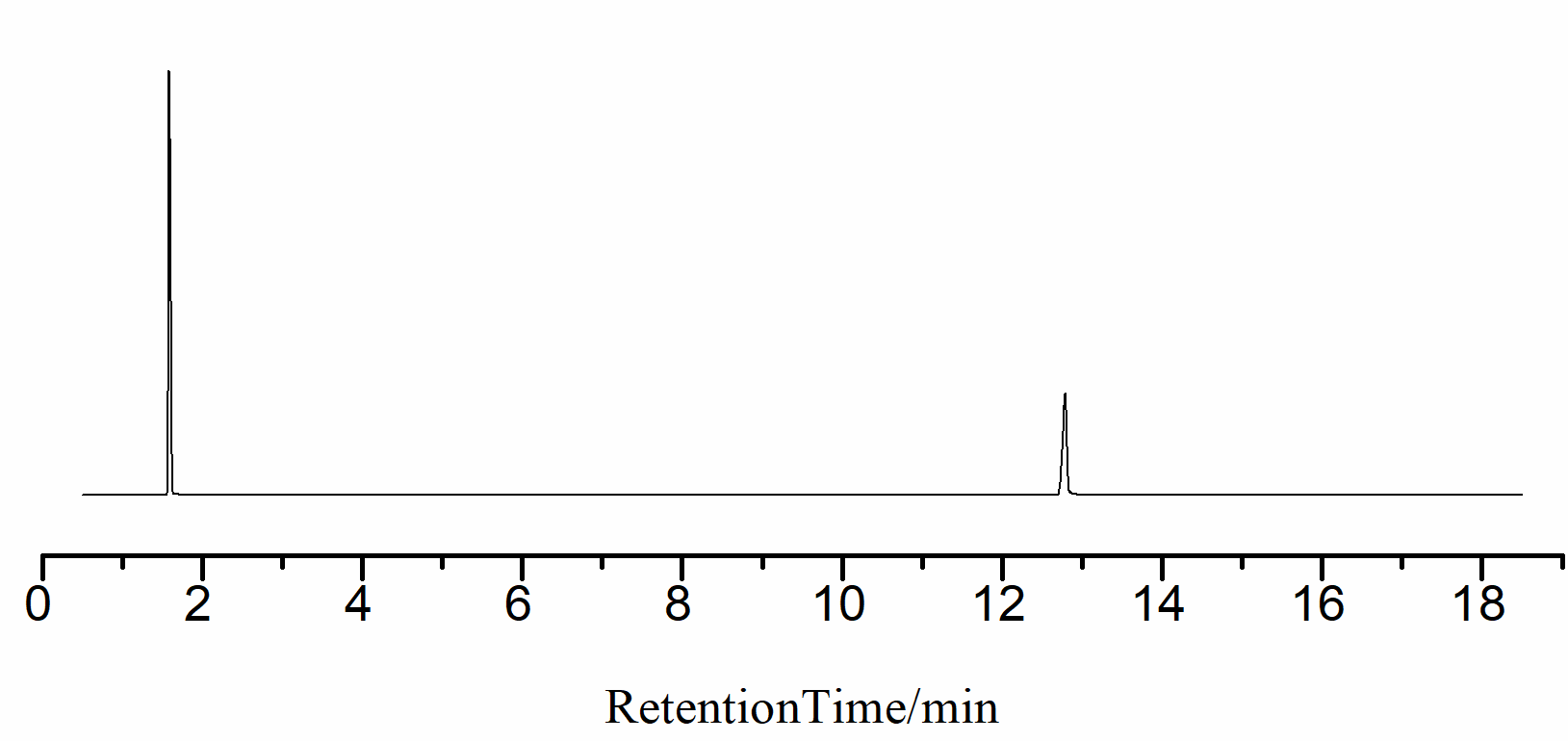


GC of cyclopentadecanolide (t=1.75 min, EtOH)


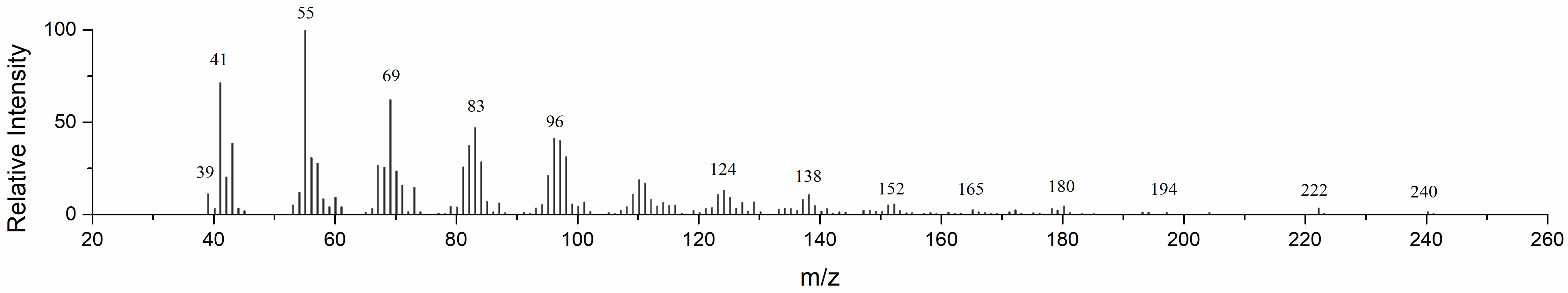


MS of cyclopentadecanolide

(EI): *m/z* = 240.2 (M^+^)

GC-MS of cyclopentadecanolide


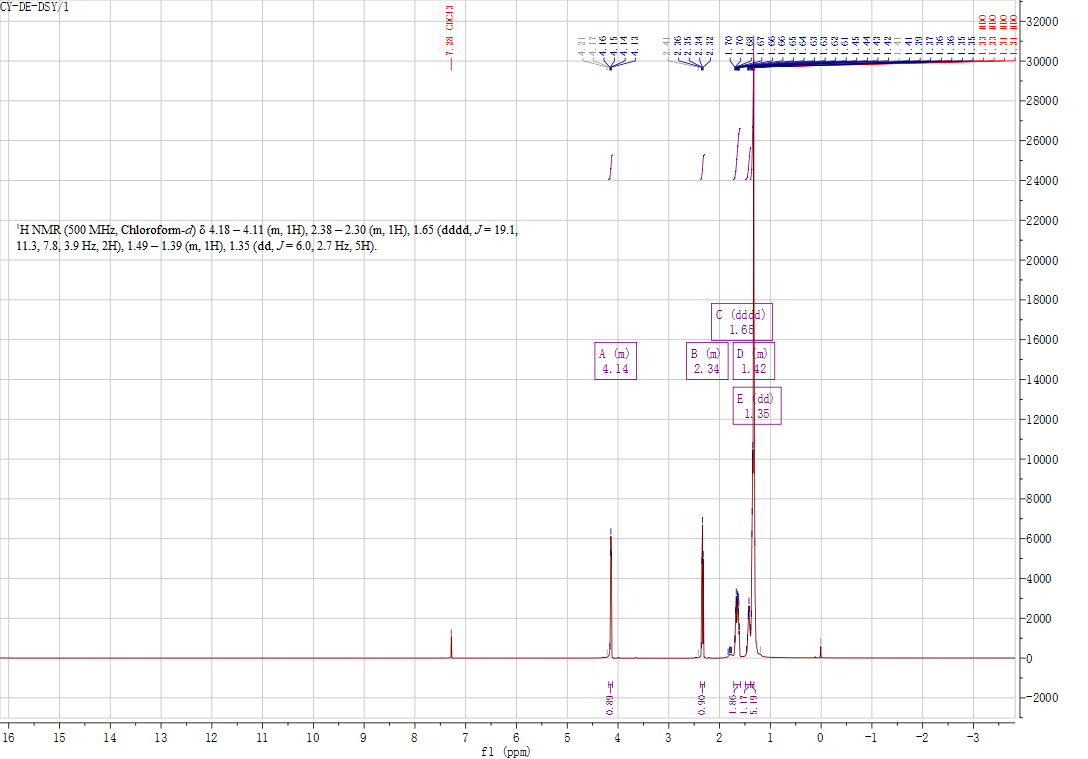


^1^H NMR of cyclopentadecanolide

^1^H NMR (500 MHz, Chloroform-*d*) δ 4.18 – 4.11 (m, 1H), 2.38 – 2.30 (m, 1H), 1.65 (dddd, *J* = 19.1, 11.3, 7.8, 3.9 Hz, 2H), 1.49 – 1.39 (m, 1H), 1.35 (dd, *J* = 6.0, 2.7 Hz, 5H).


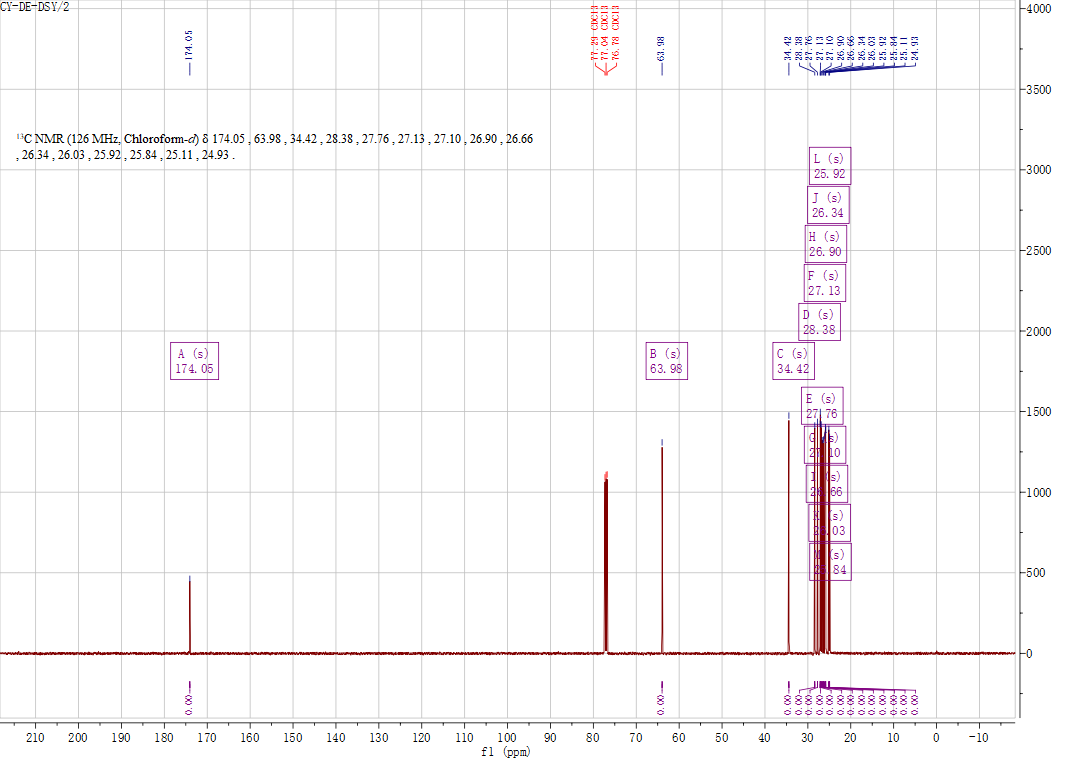


^13^C NMR of cyclopentadecanolide

^13^C NMR (126 MHz, Chloroform-*d*) δ 174.05, 63.98, 34.42, 28.38, 27.76, 27.13, 27.10, 26.90, 26.66, 26.34, 26.03, 25.92, 25.84, 25.11, 24.93.

**Cyclopentadecanone**


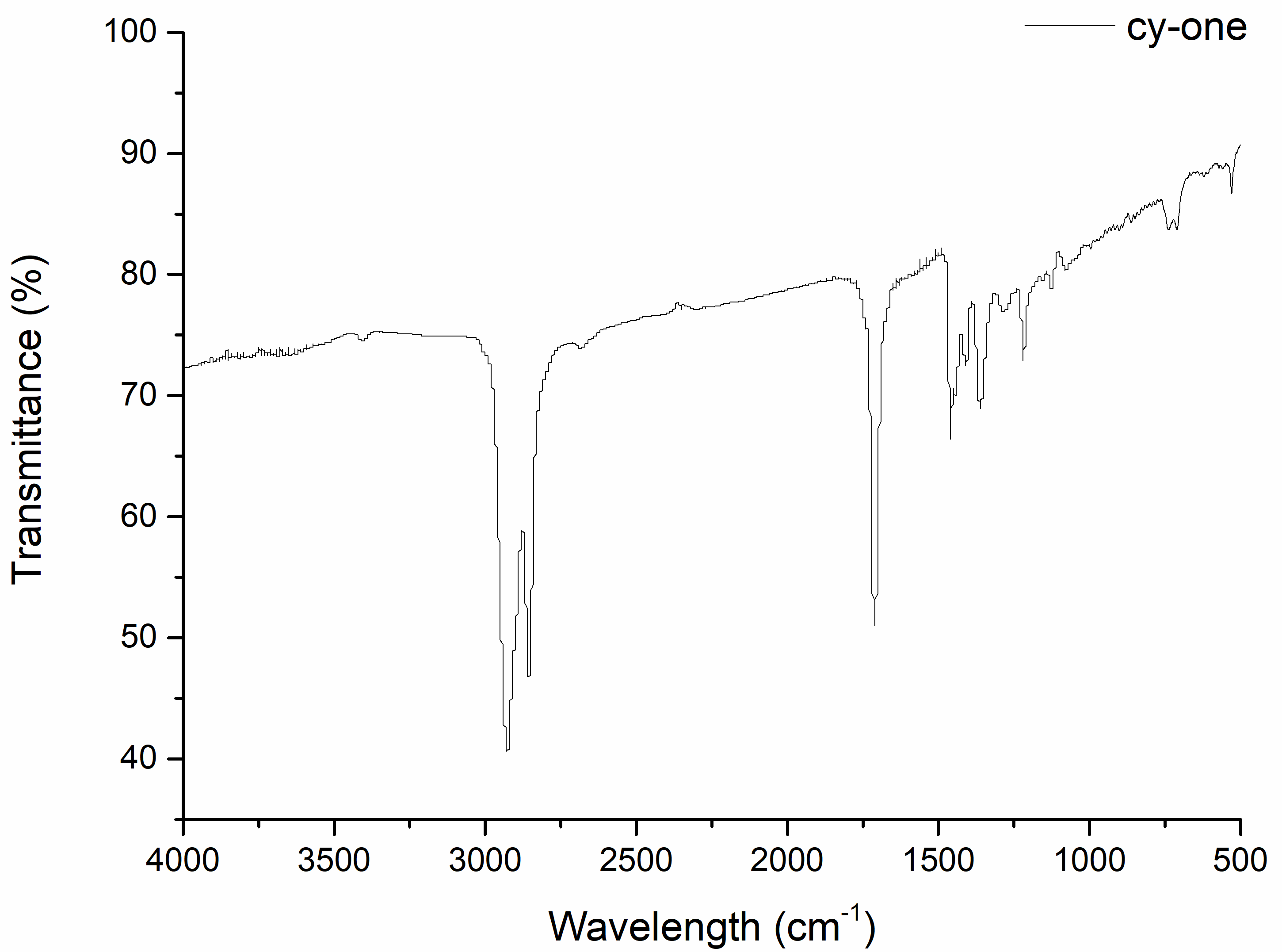


IR spectra of cyclopentadecanone

IR (KBr): 3411, 2928, 2855, 1710, 1459, 1446, 1408, 1367, 1286, 1260, 1215, 1210, 1152, 1126, 1078, 729, 720, 570 cm^-1^.


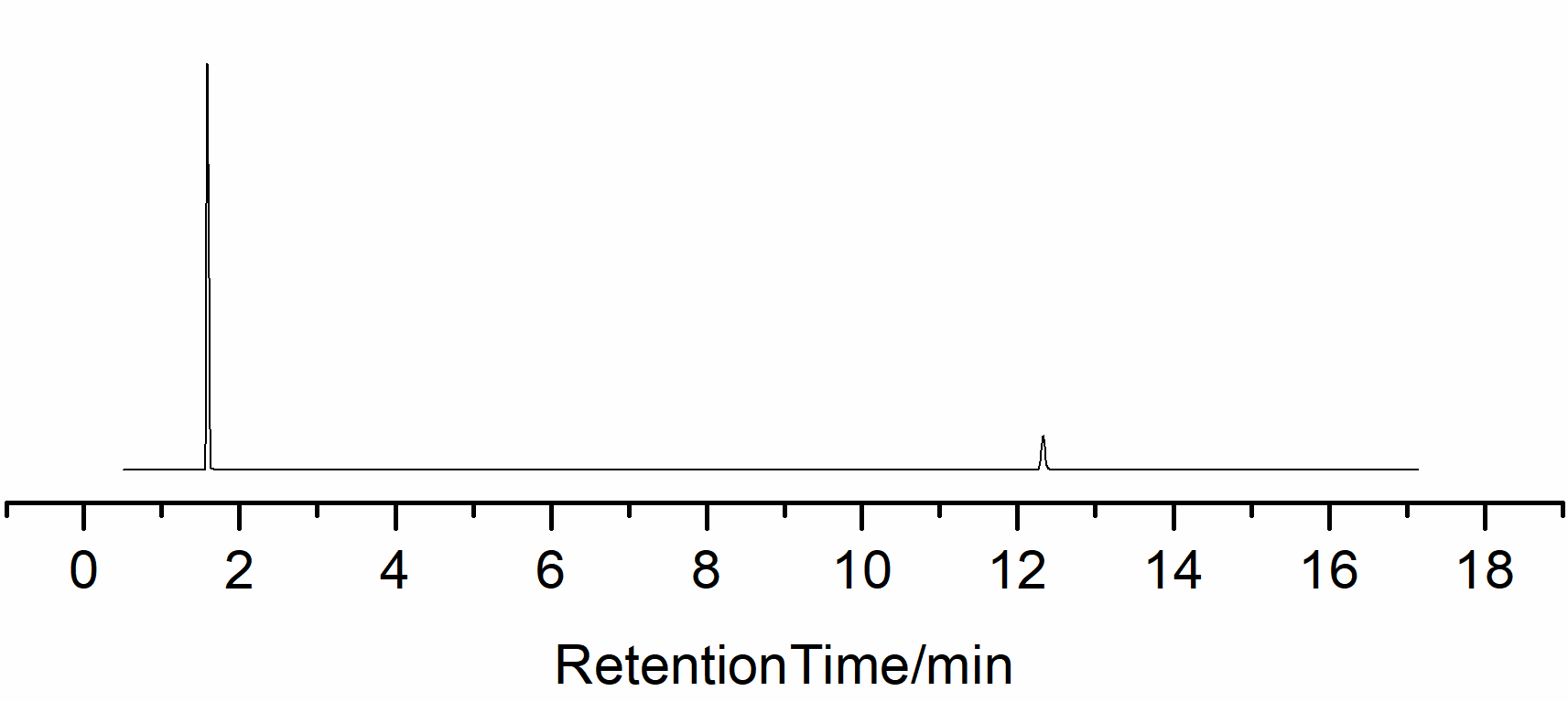


GC of cyclopentadecanone (t=1.75 min, EtOH)


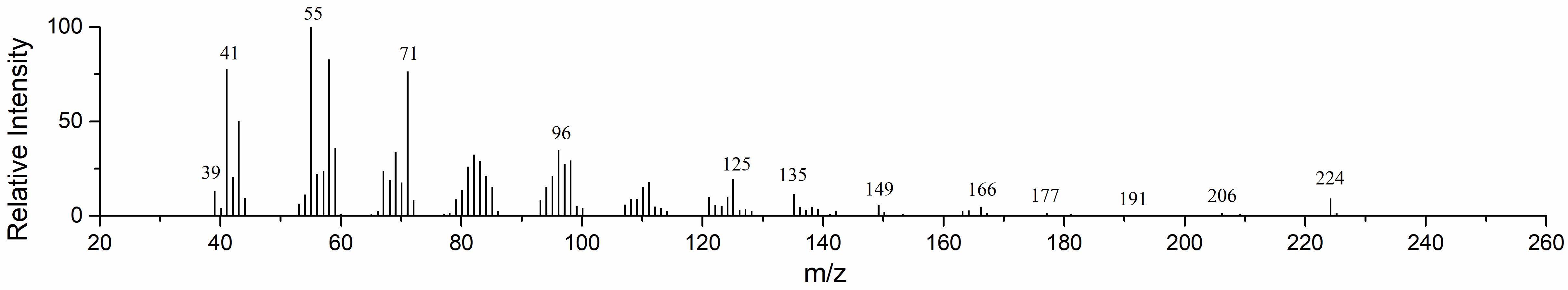


MS of cyclopentadecanone

(EI): *m/z* = 224.4 (M^+^).

GC-MS spectra of cyclopentadecanone


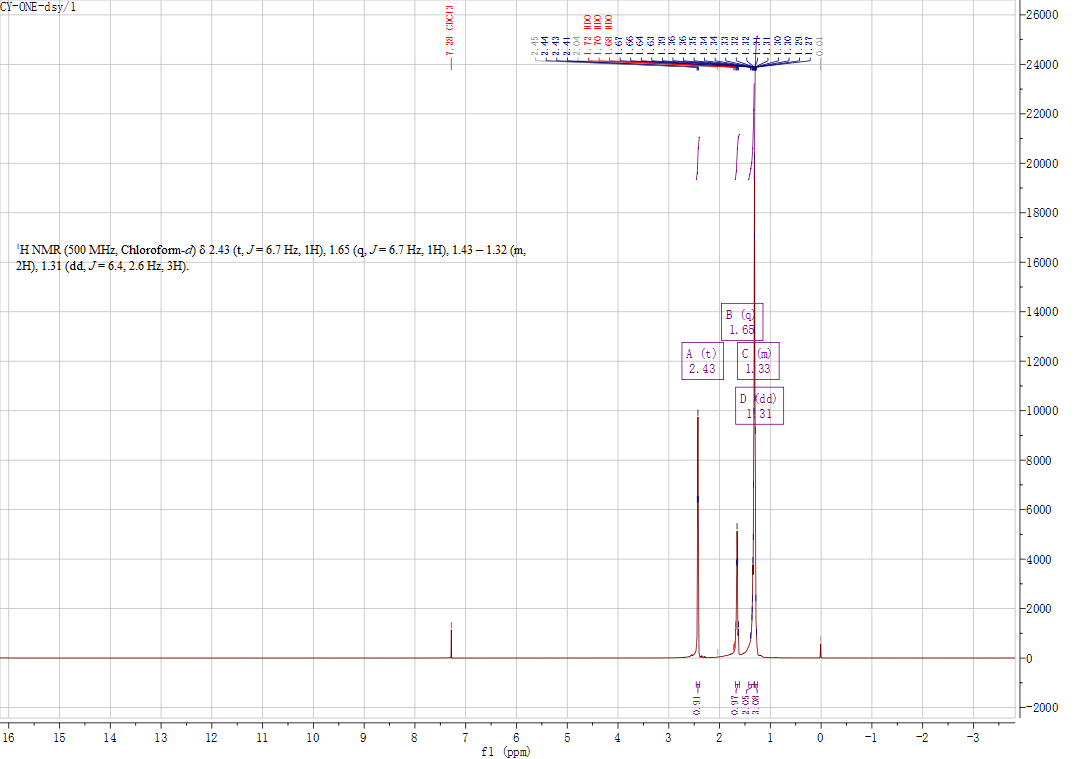


^1^H NMR of cyclopentadecanone

^1^H NMR (500 MHz, Chloroform-*d*) δ 2.43 (t, *J* = 6.7 Hz, 1H), 1.65 (q, *J* = 6.7 Hz, 1H), 1.43 – 1.32 (m, 2H), 1.31 (dd, *J* = 6.4, 2.6 Hz, 3H).


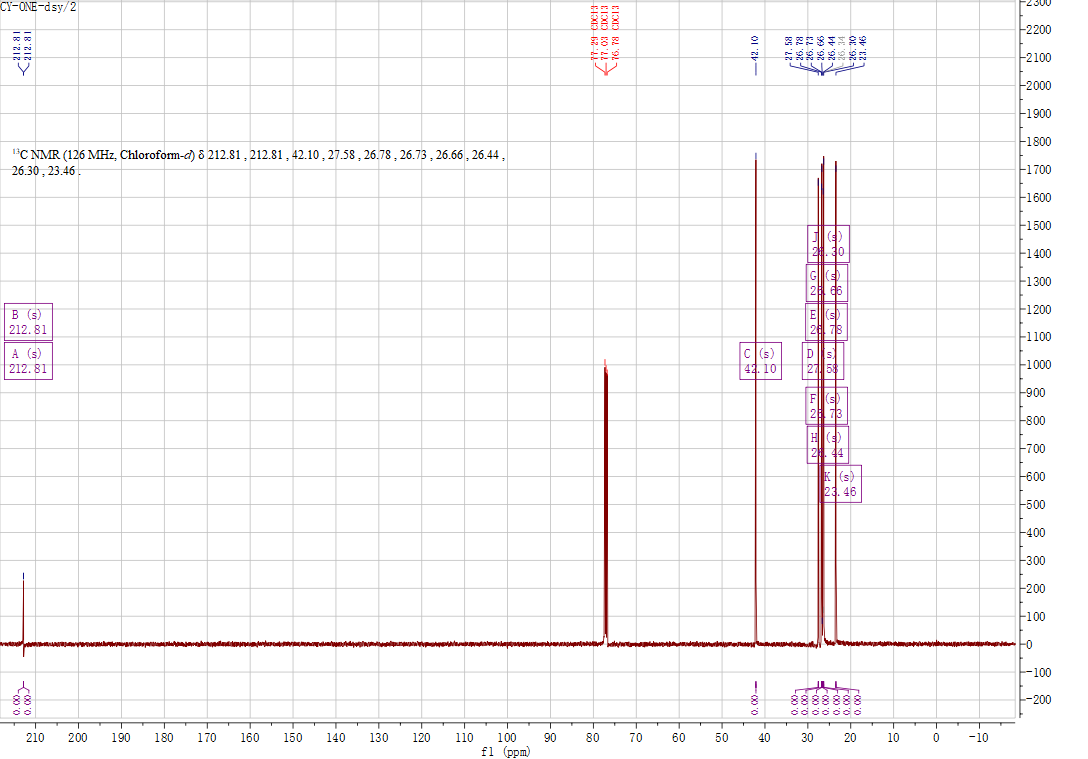


^13^C NMR of cyclopentadecanone

^13^C NMR (126 MHz, Chloroform-*d*) δ 212.81, 212.81, 42.10, 27.58, 26.78, 26.73, 26.66, 26.44, 26.30, 23.46.
